# [^18^F]Florzolotau PET for the Differential Diagnosis of Parkinsonism in Patients with Suspected 4-Repeat Tauopathies

**DOI:** 10.2967/jnumed.124.268956

**Published:** 2025-06

**Authors:** Joachim Brumberg, Nils Schröter, Ganna Blazhenets, M. Aymen Omrane, Christian Volz, Cornelius Weiller, Michel Rijntjes, Lars Frings, Sabine Hellwig, Wolfgang H. Jost, Philipp T. Meyer

**Affiliations:** 1 Department of Nuclear Medicine, Medical Center, University of Freiburg, Freiburg, Germany;; 2 Department of Neurology and Neurophysiology, Medical Center, University of Freiburg, Freiburg, Germany;; 3 Department of Psychiatry and Psychotherapy, Medical Center, University of Freiburg, Freiburg, Germany; and; 4 Parkinson-Klinik Ortenau, Wolfach, Germany

**Keywords:** tau PET, parkinsonian syndrome, 4R tauopathy

## Abstract

The second-generation tau radioligand [^18^F]florzolotau is a promising biomarker for 4-repeat (4R) tauopathies such as progressive supranuclear palsy (PSP) and corticobasal degeneration (CBD), which are difficult to disentangle clinically. Prior studies evaluating the diagnostic accuracy of [^18^F]florzolotau PET focused on highly selected patient populations (e.g., PSP–Richardson syndrome or amyloid-β–negative corticobasal syndrome). The present study assesses the diagnostic performance of [^18^F]florzolotau PET in conjunction with visual reads in a real-world clinical cohort. **Methods:** Ninety-four consecutive patients with parkinsonism and possible 4R tauopathy undergoing [^18^F]florzolotau PET for differential diagnosis were enrolled and retrospectively analyzed. The interdisciplinary consensus diagnosis based on comprehensive clinical and imaging data (most notably, [^18^F]FDG PET) served as the reference standard. [^18^F]florzolotau PET was assessed visually using predefined 4R-like and Alzheimer disease (AD)–like binding patterns (on a 4-point scale). In addition, 4R-like cases were rated with respect to the cortical–subcortical gradient of 4R-like binding. The diagnostic performance was assessed by receiver operating characteristic (ROC) analyses. **Results:** The 4R-like pattern was more prevalent and more strongly expressed (84.3%, mean score, 2.0 ± 1.1) in patients with a consensus diagnosis of PSP/CBD (joint diagnostic group of clinically likely 4R tauopathies) than in all other groups (11.6%, 0.26 ± 0.75, *P* < 0.0001). An AD-like pattern was present in all patients with a consensus diagnosis of AD (100%, 2.5 ± 0.9) and at high frequency, albeit with lower magnitude, in all other patient groups (67.4%, 1.2 ± 1.1, *P* < 0.01). ROC analysis for the 4R-like pattern (PSP/CBD vs. all other patients) yielded an area under the ROC curve (AUC) of 0.87 (sensitivity, 84.3%; specificity, 88.4%). The diagnostic performance of [^18^F]florzolotau PET did not change when also considering the AD-like pattern (AUC, 0.88; logistic regression, factor AD-like pattern; *P* = 0.53) or excluding all cases with AD (AUC, 0.86). The presence of corticobasal syndrome in patients with 4R-like binding was strongly associated with preferentially cortical binding (AUC, 0.89). **Conclusion:** Based on a real-world population of patients with parkinsonism, we demonstrate that simple visual evaluation of [^18^F]florzolotau PET by an a priori–defined 4R-like binding pattern allows highly accurate identification of patients with a consensus diagnosis of PSP/CBD. Thus, [^18^F]florzolotau PET is a promising biomarker for differential diagnosis of neurodegenerative parkinsonian syndromes.

## Visual Abstract

**Figure:**
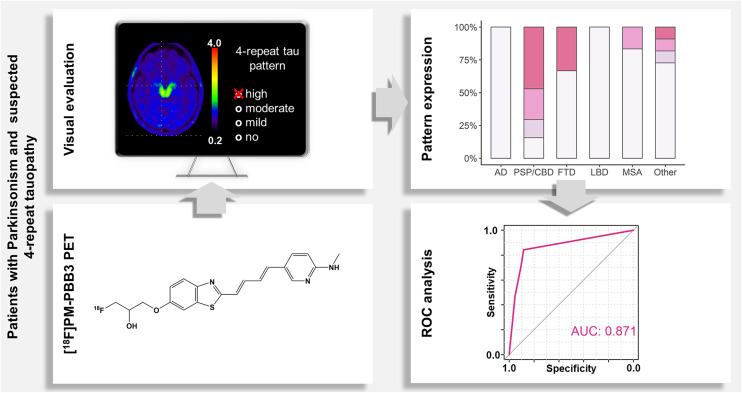

Second-generation tau PET radioligands, which bind to 4-repeat (4R) tau isoforms in progressive supranuclear palsy (PSP) and corticobasal degeneration (CBD), in addition to mixed 3-repeat and 4R tau in Alzheimer disease (AD), may enable tau imaging for differential diagnosis of parkinsonism. The recently proposed ligands 2-(2-([^18^F]fluoro)pyridin-4-yl)-9*H*-pyrrolo[2,3-b:4,5c′]dipyridine ([^18^F]PI-2620) and 1-[^18^F]fluoro-3-((2-((1*E*,3*E*)-4-(6-(methylamino)pyridine-3-yl)buta-1,3-dien-1-yl)benzo[*d*]thiazol-6-yl)oxy)propan-2-ol ([^18^F]PM-PBB3, official generic name: florzolotau (18F) or florzolotau) proved to be promising tau biomarkers in patients with suspected 4R tauopathies in several studies, including patients with a clinical diagnosis of PSP (1,2) or amyloid-β (Aβ)–negative corticobasal syndrome (CBS) (3,4). In patients with CBS, AD ranks as the third most common underlying pathology (∼13%–20%) after CBD (∼24%–55%) and PSP (∼17%–31%) (5,6). Therefore, most CBS cases that are Aβ-negative are likely due to either CBD or PSP, which are hard to disentangle clinically (7). The patterns of cerebral [^18^F]florzolotau binding in groups of patients with PSP and Aβ-negative CBS significantly differed from the pattern found in AD patients (i.e., closely reflecting neurofibrillary tangle stages in AD) (8). In turn, the reported patterns in both aforementioned groups are similar: patients with PSP and Aβ-negative CBS exhibit increased subcortical binding in the mid-brain, thalamus, and globus pallidus, and—to varying degrees—cortical binding in the pre- and postcentral gyrus, supplementary motor area, and subcortical white matter (1,3,8). These observations are consistent with the clinicopathologic overlap between PSP and CBD in terms of clinical syndromes and the distribution of 4R tau neuropathology with varying degrees of subcortical or cortical predominance (7,9,10).

PSP and CBD have various clinical presentations (8 predominance types were defined in recent PSP criteria) (5,7,11). In addition, they show a diverse temporal evolution across syndromes with a loss of phenotypic diversity and convergence (e.g., to PSP–Richardson syndrome [PSP-RS], with the highest tau load and fastest progression in the case of PSP), including aphasia and dementia (12–14). Thus, a focus on (advanced) subsets of this spectrum and comparison of them to well-defined control groups—for example, PSP-RS versus healthy control, AD, and α-synucleinopathy groups (2) or PSP versus α-synucleinopathy groups (1)—neglects the clinicopathologic overlap between PSP and CBD and carries a high risk of bias (e.g., overestimating diagnostic accuracy because of selective patient inclusion).

Thus, the aim of the present study was to assess the diagnostic performance of [^18^F]florzolotau PET for identifying 4R tauopathies in a representative clinical cohort of patients with parkinsonism and suspected underlying 4R tauopathy. To improve the accuracy of the reference diagnosis in the absence of autopsy data, we used an interdisciplinary consensus diagnosis incorporating extensive clinical, laboratory, and imaging data. The latter most notably included [^18^F]FDG PET in virtually all patients, which has been demonstrated to be of high diagnostic value in parkinsonian syndromes and allows accurate distinction not only between AD-CBS and Aβ-negative CBS but also between pathologically verified PSP and CBD (15–17). To meet the aforementioned challenges, we first analyzed the accuracy of [^18^F]florzolotau PET for diagnosing PSP/CBD (joint diagnostic group of clinically likely 4R tauopathies), which was then stratified by the presence or absence of clinical CBS.

## MATERIALS AND METHODS

### Patients

This retrospective study includes 94 consecutive patients who underwent [^18^F]florzolotau PET for differential diagnosis of parkinsonism at a tertiary medical center (internal referral for PET from neurologists and psychiatrists or external referral from a specialized movement disorder hospital). The indication for [^18^F]florzolotau PET was given by the clinical suspicion of an underlying 4R tauopathy. The interdisciplinary consensus diagnosis combining comprehensive clinical and imaging data (among others, [^18^F]FDG PET in 90 cases) served as the reference standard and was obtained by an interdisciplinary expert panel according to current diagnostic criteria (11,18–22) and a standardized procedure (23,24) masked to [^18^F]florzolotau PET results (details are in the supplemental materials [supplemental materials are available at http://jnm.snmjournals.org]). The a priori–defined diagnostic categories were PSP/CBD (subsuming PSP, its predominance types, and CBD-CBS), Lewy body diseases (LBDs; subsuming Parkinson disease [PD], PD with dementia [PDD], and dementia with Lewy bodies [DLB]), multiple system atrophy (MSA; with predominant parkinsonism or cerebellar ataxia), AD (including AD variants; e.g., AD-CBS and logopenic variant primary progressive aphasia), and frontotemporal dementia (FTD; i.e., behavioral variant FTD, semantic variant primary progressive aphasia, and nonfluent variant primary progressive aphasia). Patients, who did not fulfill the criteria of any of these categories were classified as other. PSP/CBD was further stratified into PSP/CBD with and without CBS. CBS was defined clinically, requiring 1 cortical and 1 movement disorder sign (11). If available, symptom duration, Hoehn and Yahr stages, and Unified Parkinson Disease Rating Scale part III, Mini-Mental State Examination, and Montreal Cognitive Assessment scores were used for correlational analyses. For this purpose, Montreal Cognitive Assessment scores were converted into Mini-Mental State Examination scores (25). All patients gave written informed consent to undergo [^18^F]florzolotau PET as part of their clinical work-up and to retrospective analysis of their data. The institutional review board approved the study (27/18 and 100/19). It was conducted in accordance with the principles of the Declaration of Helsinki and its later amendments.

### PET Analysis

PET acquisition and processing are described in detail in the supplemental materials. All patients underwent a 20-min PET scan 90 min after injection of 356 ± 21 MBq of [^18^F]florzolotau on a fully digital Vereos PET/CT scanner (Philips Healthcare) (8). Parametric [^18^F]florzolotau SUV ratio images scaled to the inferior cerebellar cortex were used for visual analysis. On the basis of prior studies (3,8), we defined the 4R-like pattern as locally increased uptake using a standardized color scale (rainbow; SUV ratio range, 0.2–4.0) in the mid-brain, thalamus, globus pallidus, pre- and postcentral gyrus, and supplementary motor area (with possible subcortical or cortical predominance, as described later). The AD-like pattern was defined as locally increased uptake using the same standardized color scale in mesial temporal and neocortical regions (i.e., in temporal, parietal, and frontal cortices and the posterior cingulate gyrus or precuneus) with relative sparing of subcortical structures (except the caudate nucleus) and the primary sensorimotor and visual cortex (3,8). Two experienced raters and 1 less experienced rater (all masked to the reference diagnoses and clinical information) rated [^18^F]florzolotau PET scans on 30 transaxial slices covering the entire brain. The raters classified [^18^F]florzolotau PET scans according to the presence and expression of AD-like and 4R-like [^18^F]florzolotau binding patterns using a 4-point scale (0, no; 1, mild; 2, moderate; 3, strong). In presence of a 4R-like pattern, raters subsequently assessed the gradient of the 4R-like pattern by rating the dominance of subcortical (score 0), or cortical (score 2) [^18^F]florzolotau binding. Comparable binding in subcortical and cortical structures was considered balanced (score 1). After independent evaluation of individual [^18^F]florzolotau PET scans, the raters reached a consensus score.

### Statistical Analysis

Analyses were performed with R software (version 4.3.0; R Project for Statistical Computing). Differences between groups were assessed with ANOVA (followed by a Tukey honest significant difference test) or a Kruskal–Wallis, Wilcoxon rank-sum, or χ^2^ test, as appropriate. Interrater reliability was evaluated with Cohen-weighted κ. We used receiver operating characteristic (ROC) analyses to assess the performance of the consensus score of [^18^F]florzolotau PET binding patterns and thus discriminate PSP/CBD (combined, as well as the groups with and without CBS) from other patient groups, as well as to discriminate PSP/CBD with CBS from PSP/CBD without CBS using the area under the ROC curve (AUC) as an outcome measure. The optimal cutoff was evaluated using the Youden index. ROC AUCs were compared with the Delong test.

## RESULTS

### Patient Characteristics

**FIGURE 1.**
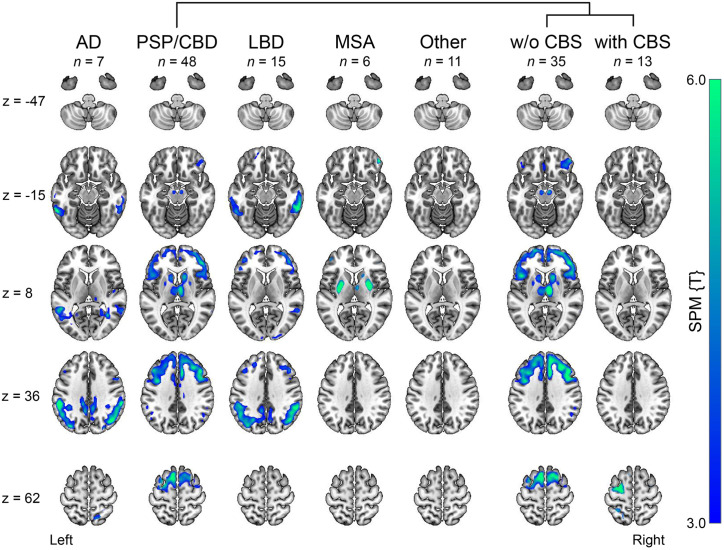
Groupwise patterns of cerebral hypometabolism. [^18^F]FDG PET scans supporting interdisciplinary consensus diagnosis were compared with 13 healthy controls (false discovery rate–corrected *P* < 0.05, cluster extent *k* > 100 voxels). Significant clusters are overlaid on MRI template. [^18^F]FDG PET scans were available for all patients except for 4 classified as AD or PSP/CBD. Patients with FTD (*n* = 3) and LBD subgroups without and with dementia are shown in Supplemental Figure 2. SPM {T} = statistical parametric mapping T value; w/o = without; z = slice number.

Patient flow is shown Supplemental Figure 1. Patient characteristics are summarized in Table 1. Clinical consensus diagnoses were as follows: 51 PSP/CBD (16 PSP-RS, 13 PSP with predominant parkinsonism, 6 PSP/CBS, 4 PSP with predominant frontal presentation, 5 PSP without clear predominance type, and 7 CBD-CBS), 15 LBD (7 PD, 4 PDD, and 4 DLB), 6 MSA (all MSA with predominant parkinsonism), 8 AD (among them, 2 AD-CBS), 3 FTD (1 behavioral variant FTD and 2 nonfluent variant primary progressive aphasia), and 11 other (7 nonneurodegenerative disease, 2 subcortical leukoencephalopathy, 1 amyotrophic lateral sclerosis, and 1 Creutzfeldt–Jakob disease). Figure 1 and Supplemental Figure 2 depict the typical disease-specific patterns of cerebral glucose metabolism in the different disease groups (including stratification by dementia of LBD and individual FTD cases, given their low number), supporting the validity of the interdisciplinary consensus diagnosis. There were no differences between groups for age at PET (*F*_5,88_ = 1.5, *P* = 0.21) and symptom duration (*F*_5,85_ = 1.4, *P* = 0.24; albeit longer in LBD). Average Mini-Mental State Examination scores indicated mild dementia in LBD, AD, and FTD patients without significant differences compared with other groups (*F*_5,68_ = 1.6, *P* = 0.17). Overall motor impairment, as assessed by the Hoehn and Yahr scale, was on a trend level higher in MSA (strongly impaired) than in patients with PSP/CBD (moderate impairment; *F*_3,63_ = 3.3, *P* = 0.03; Tukey honest significant difference test, MSA vs. PSP/CBD, *P* = 0.08). The rank order was similar on a more detailed assessment with Unified Parkinson Disease Rating Scale part III without significant group differences (*F*_4,49_ = 0.25, *P* = 0.91).

**TABLE 1.** Demographic and Clinical Characteristics

Characteristic	AD	PSP/CBD	FTD	LBD	MSA	Other	*P* for ANOVA/ χ^2^ test
Patients (*n*)	8	51	3	15	6	11	—
Sex (M/F)	1/7	19/32	2/1	9/6	2/4	8/3	0.07
Age (y)	64.2 ± 10.6	71.5 ± 7.8	71.7 ± 6.1	72.4 ± 7.1	68.3 ± 6.6	70.9 ± 6.8	0.21
Symptom duration (y), *n* = 91	2.8 ± 2.8	3.2 ± 2.3	2.0 ± 1.4	5.1 ± 5.7	3.9 ± 1.9	2.3 ± 1.7	0.24
Hoehn and Yahr, *n* = 69	2.0*	3.0 ± 0.8	2.0*	3.0 ± 0.6	3.8 ± 0.8	3.8 ± 1.1	0.03
UPDRS part III, *n* = 55	39.5 ± 17.7	47.0 ± 24.9	69.0*	42.6 ± 12.7	51.8 ± 7.8	50.0 ± 12.2	0.91
MMSE, *n* = 74	23.3 ± 3.6	25.7 ± 3.5	23.7 ± 3.1	24.0 ± 4.6	27.5 ± 0.6	26.4 ± 1.6	0.18

* Excluded from ANOVA because of availability in only 1 patient.

UPDRS = Unified Parkinson Disease Rating Scale; MMSE = Mini-Mental State Examination.

Data are number or mean ± SD.

### Visual Ratings of [^18^F]Florzolotau PET Binding

**FIGURE 2.**
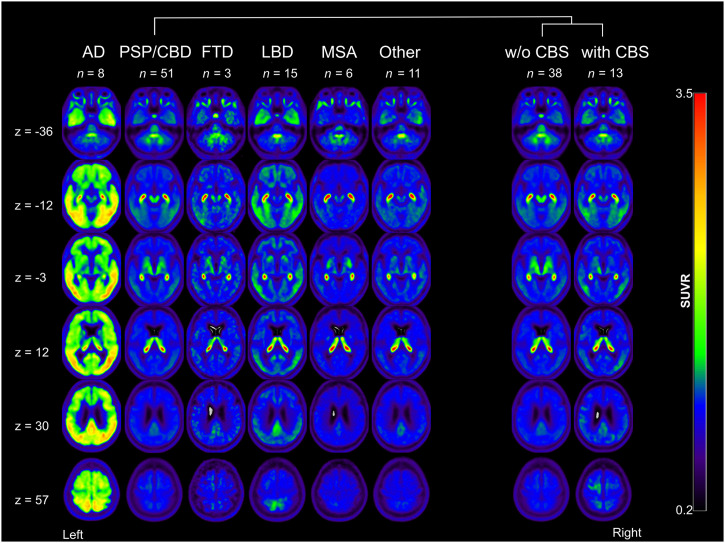
Group averaged [^18^F]florzolotau PET data. Each column shows 6 representative transaxial slices of disease group. SUVR = SUV ratio; w/o = without; z = slice number.

**FIGURE 3.**
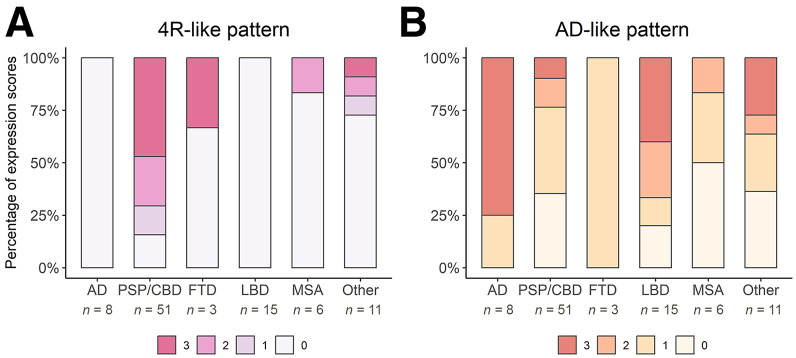
Percentages of visual pattern ratings. (A and B) Proportional 4R-like (A) and AD-like (B) pattern expression by each diagnostic group.

Figure 2 depicts representative transaxial slices of average [^18^F]florzolotau PET images of each group. Figure 3 and Supplemental Table 1 illustrate the proportion of visual ratings for 4R-like or AD-like patterns in the different patient groups. The 3 raters reached substantial agreement for the classification of 4R-like (κ range, 0.78–0.97) and AD-like (κ range, 0.88–0.96) patterns (higher agreement between more experienced raters, 0.97 vs. 0.84).

The expected 4R-like pattern from the literature was replicated by the present sample (Fig. 2). This 4R-like pattern was more highly prevalent (84.3%) and expressed (mean score, 2.0 ± 1.1) in the PSP/CBD group than in all other disease groups (pooled prevalence, 11.6%; mean score, 0.26 ± 0.75; *P* < 0.0001 compared with the 4R group). The pattern was observed in most PSP/CBD cases with moderate to strong expression (12/51 [23.5%] and 24/51 [47.1%], respectively), whereas only few patients with PSP/CBD showed no or mild pattern expression (8/51 [15.7%] and 7/51 [13.7%], respectively). Consensus ratings did not significantly differ between patients without CBS (2.2 ± 1.1) and with CBS (1.5 ± 1.2, *P* = 0.08). However, the gradient of binding was significantly different: patients with PSP/CBD without CBS (PSP, according to consensus diagnoses) showed more pronounced subcortical binding (gradient rating, 0.3 ± 0.7), whereas those with CBS exhibited more cortical binding (gradient rating, 1.6 ± 0.5; *P* < 0.0001).

In turn, the 4R-like pattern was not detected in any AD and LBD cases. One of 3 (33.3%) FTD cases was rated to show a strong 4R-like pattern (not present in the remaining 2 cases), whereas only 1 of 6 (16.7%) MSA cases showed moderate 4R-like pattern expression (not present in the remaining 5 cases). Three of 11 patients with other diagnoses were rated as showing a mild, moderate, and strong 4R-like pattern (final diagnoses of nonneurodegenerative disease, subcortical leukoencephalopathy, and nonneurodegenerative disease, respectively).

The AD-like pattern was also well replicated in the present sample (Fig. 2). Consensus ratings indicated strong and mild AD-like pattern expression in 6 of 8 (75.0%) and 2 of 8 (25.0%) patients, respectively (i.e., prevalence of 100% and mean score of 2.5 ± 0.9 in the AD group). The AD-like pattern was also frequently present in other patient groups (pooled prevalence, 67.4%), albeit at a lower magnitude (mean score, 1.2 ± 1.1; *P* < 0.01 compared with the AD group): moderate and strong AD-like pattern expression was rated in a substantial fraction of LBD cases (moderate, 4/15 [26.7%], 3/7 PD and 1/4 PDD; strong, 6/15 [40.0%], 2/4 PDD and 4/4 DLB) and a few PSP/CBD cases (moderate, 7/51 [13.7%]; strong, 5/51 [9.8%]; at higher frequency in patients with CBS [6/13] than without CBS [6/38]). The remaining LBD (5/15 [33.3%], 4 PD and 1 PDD), PSP/CBD (39/51 [76.5%]), FTD (3/3), and MSA (except 1/6 with moderate pattern expression) patients showed no or mild AD-like pattern expression (Fig. 3). In patients with other diagnoses, AD-like pattern ratings were roughly equally distributed across the 4 grades (Fig. 3).

### Discriminative Analysis

The ROC analysis for the consensus score of the 4R-like pattern (PSP/CBD vs. all other patients) revealed AUC of 0.87 (95% CI, 0.80–0.94; range of the 3 raters, 0.78–0.87; highest in experienced raters). The optimal threshold for delineating PSP/CBD from all other disease groups was a score of at least 1 (i.e., at least mild 4R-like pattern), leading to a sensitivity of 84.3% (71.4%–93.0%) and a specificity of 88.4% (74.9%–96.1%). The positive predictive value was 89.6% (77.2%–95.5%), and the negative predictive value was 82.6% (68.8%–93.9%). When stratified in PSP/CBD without and with CBS, ROC analyses yielded comparable AUC (0.89; 95% CI, 0.82–0.96; and 0.82; 95% CI, 0.69–0.95, respectively; Delong test, *P* = 0.39). When focusing on proper parkinsonian syndromes (i.e., PSP/CBD vs. LBD and MSA), the ROC AUC was also largely unchanged (0.91; 95% CI, 0.85–0.97; *P* = 0.46).

The AD-like pattern did not support the distinction of patients with PSP/CBD alone (AUC, 0.36; range, 0.25–0.47), nor did it improve the distinction in combination with the 4R-like pattern (logistic regression; AUC, 0.88; range, 0.81–0.95; factor AD-like pattern; *P* = 0.53). Conversely, exclusion of AD cases from the aforementioned analysis concerning the discrimination of PSP/CBD did not affect overall diagnostic performance (AUC, 0.86; range, 0.78–0.94; *P* = 0.83). Despite the limited AD sample size and the diversity of other diagnoses, the AD-like pattern effectively distinguished the AD group from all other disease categories, achieving AUC of 0.82 (0.68–0.96).

Finally, the rating of the binding gradient of the 4R-like pattern allowed an accurate distinction between patients with and those without CBS in PSP/CBD patients who exhibited a 4R-like pattern (43/51; ROC AUC, 0.89; ROC AUC range, 0.80–0.98). In addition, 27 of 33 (81.8%) patients without CBS showed subcortical dominant binding, whereas all patients with CBS exhibited balanced or cortical dominant binding.

## DISCUSSION

The present study was conducted in a real-world sample of patients with parkinsonism that reflects the expected indication for tau PET in the clinical routine. It demonstrates that a visually rated 4R-like binding pattern on [^18^F]florzolotau PET allows the identification of patients with PSP/CBD with high sensitivity and specificity. In addition, a gradient of the 4R-like pattern toward cortical binding was highly associated with the presence of CBS in patients with a 4R-like pattern. Although the AD-like pattern of [^18^F]florzolotau binding as a marker of (early) AD copathology or possibly primary age-related tauopathy was common in this representative clinical cohort, it did not significantly affect the diagnostic performance of the 4R-like pattern.

Visual evaluation of the 4R-like pattern revealed ROC AUC of 0.87 for the discrimination of PSP/CBD from all other groups, which remained largely unchanged when focusing on proper parkinsonian syndromes (0.91) or excluding all AD patients (0.86). This aligns well with previous findings, which showed AUC between 0.87 and 0.94 for a volume-of-interest–based approach to separate PSP from α-synucleinopathies with [^18^F]florzolotau PET (1), and it suggests that the high specificity in this representative sample (88%) is not driven by easy identification of AD-related syndromes. This study validates promising earlier data in a larger clinical cohort that includes not only or predominantly patients with probable or later-stage PSP or PSP-RS and somewhat artificial controls (e.g., AD or healthy controls). Instead, the present sample appropriately covers the entire spectrum of syndromes and diagnoses that would be expected in parkinsonism with possible 4R tauopathies based on clinicopathologic studies (as explained in the introduction). Moreover, current estimates for sensitivity (84%) and specificity (88%) of the 4R-like pattern on [^18^F]florzolotau PET suggest an advantage in comparison to the second-generation ligand [^18^F]PI-2620, which allows detection of PSP-RS with high sensitivity (85%, at 77% specificity), whereas non–Richardson-type PSP (65%) and Aβ-negative CBS (65%) were detected with noticeably lower sensitivity (2,4). The mean disease duration of patients with PSP enrolled in the aforementioned studies (1,2) was longer (4.1 and 3.8 y) than that of the current PSP/CBD group (3.2 y), highlighting the possible value of [^18^F]florzolotau for earlier diagnosis. Moreover, the simple acquisition protocol and the easy-to-implement workflow for visual image analysis provide practical advantages compared with [^18^F]PI-2620 (60-min acquisition and kinetic modeling) (2). Cases of FTD and MSA with 4R-like pattern expression (1 patient each) are in line with the previous literature and may be related to the occurrence of 4R tau pathology in nonfluent variant primary progressive aphasia and behavioral variant FTD (26,27) or to misclassification because of possible off-target binding of [^18^F]florzolotau in MSA (28).

Previously described binding patterns for clinically likely 4R tauopathies and AD were well replicated in the present cohort (1,3,8). This also applies to the binding gradient of the 4R-like pattern. Most patients correctly assigned to the PSP/CBD group had no CBS. These patients showed subcortical dominant binding in 81.8%. However, all cases with CBS (clinical consensus diagnosis of PSP-CBS and CBD-CBS with an 4R-like pattern score ≥ 1, 5 cases each) showed predominantly cortical binding (60.0%) or balanced binding between cortical and subcortical structures (40.0%). This was also the case in 6 patients with PSP/CBD without clinical CBS (balanced, 1 PSP-RS; cortical predominance, 3 PSP without a clear predominance type and 2 PSP with predominant frontal presentation). Although a comparably severe 4R tau pathology in cortical areas can be expected in PSP with predominant frontal presentation (9), this may imply that cortical 4R tau pathology precedes the clinical manifestation of cortical symptoms and CBS in the other cases. Therefore, a possibly predictive value of the binding gradient requires further research. Overall, the results confirm the approach of the current study to consider the binding of [^18^F]florzolotau not only as a diagnostic marker for a subset such as PSP-RS but also as a marker for the entire group of 4R tauopathies.

Although the 4R-like pattern showed high specificity for PSP/CBD and was not observed in AD and LBD cases, patients exhibited an AD-like pattern of varying degrees across all diagnostic groups. Besides in AD and in line with a previous study (29), strong AD-like pattern expression was predominantly observed in patients with PDD and DLB. Although Aβ status was not systematically evaluated in this study, this finding suggests the actual presence of AD-type copathology, which frequently occurs besides α-synuclein pathology in PDD and DLB (30). Given the substantial prevalence of mild AD-like pattern expression across all groups, it is tempting to speculate that this may be caused by primary age-related tauopathy (31). However, because of the unknown amyloid status of most patients (*n* = 72), early AD copathology in elderly subjects as the origin of this pattern expression may be possible. Overall, our additional analyses (i.e., logistic regression analysis of combined AD and 4R patterns and exclusion of AD patients) imply that the frequent occurrence of the AD-like pattern has no relevant effect on diagnostic accuracy in the present clinical context.

Previous studies with [^18^F]florzolotau selected patients with parkinsonism according to their established clinical diagnoses (1,3,28,29). In contrast, consecutive enrollment for this study includes patients with uncertain diagnoses, including less typical presentations, mixed pathologies, and comorbidities. Consequently, the present cohort reflects the clinical reality and the need for more advanced diagnostic options in unclear cases, what we consider a particular strength of this study. We cannot exclude that the lack of healthy controls may have affected the results. However, according to prior studies (1,3,8), we do not expect controls to show a stronger 4R-like pattern than the present non-PSP/CBD groups, so this effect should be minimal. Another strength of the present study is the masked, interdisciplinary consensus diagnosis used as a reference standard, because it incorporates comprehensive clinical data and extensive auxiliary examinations such as cognitive assessment, cerebrospinal fluid markers, and imaging (21,32). Among the biomarkers, the availability of [^18^F]FDG PET in almost all patients (96%) provides highly valuable information for the differentiation of neurodegenerative parkinsonian syndromes (Fig. 1) (15). Although autopsy data suggest that [^18^F]FDG PET can discriminate between PSP and CBD (17), further confirmatory data are needed, and the distinction between PSP and CBD by [^18^F]FDG PET in the present study (not relevant for the primary analysis) has to be contemplated with caution.

This study has several limitations. Despite the overall large study cohort, the non-4R subgroups are rather small. This and the lack of comprehensive disease-specific clinical scores in all subjects prohibits in-depth clinicoimaging correlations. Another limitation of the retrospective study design is the inherent risk of bias. We used a securely masked analysis approach based on extensive clinical information, as previously established (23,24). Still, despite all efforts to enhance the validity of our clinical consensus diagnosis, current results need further validation by autopsy data. Two [^18^F]florzolotau PET readers were also involved in the interdisciplinary consensus diagnosis. Therefore, the frequency of [^18^F]florzolotau PET patterns may have been known to the experts to some degree, but we consider the risk of bias to be low. On the one hand, visual ratings were done several weeks before the consensus diagnosis. On the other hand, we used an ordinal (not binary) rating scale, and the diagnostic threshold was determined even later by ROC analysis. The use of the inferior cerebellar cortex as the reference region for [^18^F]florzolotau PET might be seen as critical, because the cerebellum is not devoid of tau aggregation in PSP (10). However, at this stage, we consider the widely established cerebellar reference (1,3,8,28,29) to be a suitable option.

## CONCLUSION

Based on a real-world population of patients with parkinsonism and possible 4R tauopathy, we demonstrate that simple visual evaluation of a priori–defined binding patterns on [^18^F]florzolotau PET allows highly accurate identification of patients with the clinical consensus diagnosis of PSP/CBD (clinically likely 4R tauopathies), including a stratification in subgroups without and with CBS. Thus, [^18^F]florzolotau PET is a promising biomarker for the differential diagnosis of neurodegenerative parkinsonian syndromes.

## DISCLOSURE

Joachim Brumberg was supported by the Berta-Ottenstein program for Advanced Clinician Scientist, University of Freiburg and honoraria outside of the submitted work (Novartis). Ganna Blazhenets was supported by Hans A. Krebs Medical Scientist Program, University of Freiburg. Nils Schröter has received honoraria outside of the submitted work (Abbvie, Novartis, and Stadapharm). No other potential conflict of interest relevant to this article was reported.

## Notes

KEY POINTS **QUESTION:** Is [^18^F]florzolotau PET a reliable diagnostic biomarker for 4R tauopathies in a real-world clinical cohort of patients with parkinsonism? **PERTINENT FINDINGS:** The 4R-like binding pattern on [^18^F]florzolotau PET provides high sensitivity and specificity for the diagnosis of patients with PSP/CBD, irrespective of possible AD copathology. **IMPLICATIONS FOR PATIENT CARE:** A simple workflow of [^18^F]florzolotau PET acquisition and analysis provides high clinical value for the differential diagnosis of neurodegenerative parkinsonian syndromes.
